# From rural Norway to high-density systems: translating nurse-led emergency models

**DOI:** 10.1080/02813432.2025.2524367

**Published:** 2025-06-23

**Authors:** Yalcin Golcuk

**Affiliations:** Department of Emergency Medicine, Faculty of Medicine, Muğla Sıtkı Koçman University, Muğla, Türkiye

Dear Editor

In light of the growing demand for equitable, scalable, and patient-centered acute care systems, the recent study by Østerbø et al. titled *“Experiences of patients with complex needs at municipal emergency outpost satellites,”* presents a timely and meaningful contribution to the evolving discourse on decentralized health service delivery. By foregrounding the voices of older adults with multimorbidity, the authors fill a critical gap in the literature, particularly regarding patient experiences within digitally supported, nurse-led acute care environments [1].

The study is particularly noteworthy for its methodologically rigorous application of semi-structured interviews and systematic text condensation, which enabled the elicitation of rich, first-person perspectives. The authors successfully illustrate how emergency outpost satellites, staffed by nurses and supported through video consultations with general practitioners, can mitigate travel burdens, enhance perceived safety, and preserve continuity of care. These findings reaffirm that trust, communication, and human presence remain indispensable, even in technologically mediated service models [2].

Drawing from our academic and clinical experience in Türkiye—a country that ranks among the highest globally in annual per capita emergency department (ED) visits—we believe this work offers valuable insights with practical relevance. In our setting, EDs report more than 2000 visits per 1000 population annually—nearly seven times higher than rates reported in developed countries such as the United States, United Kingdom, and Australia. [3]. the implementation of nurse-supported triage units with remote physician access may represent a viable solution to alleviate crowding and reduce inappropriate ED utilization. However, we also recognize the potential barriers to adaptation in middle-income contexts, including cultural expectations surrounding face-to-face physician contact, variability in digital health literacy, and regulatory constraints on nurse autonomy [4].

Although this study was conducted in the context of rural Norway, its core design features—nurse-led triage supported by teleconsultation—have clear translational relevance for high-density, overburdened systems such as Türkiye. By contrasting these divergent contexts, we aim to underscore the model’s adaptability and stimulate international dialogue on scalable acute care reform. This contrast-based perspective not only reinforces the generalizability of the study’s conclusions but also elevates the discussion to a level of global health system applicability [5].

The study also opens avenues for future research and policy innovation. Comparative evaluations of video-assisted versus in-person consultations across diverse patient populations and healthcare systems would further inform generalizability. Moreover, including the perspectives of nurses and remote physicians would enrich our understanding of operational dynamics and help shape context-specific protocols. The model described by Østerbø et al. offers a scalable framework for countries seeking to expand access to acute care without further burdening hospital-based services.

In conclusion, this study exemplifies how hybrid emergency care models can promote efficiency while preserving patient-centeredness—an increasingly critical objective in strained health systems worldwide. We thank the authors and the editorial team for advancing this important area of inquiry and offering a platform for further dialogue on the global applicability of decentralized emergency care.

## References

[1] Østerbø T, Hovland G, Ytrehus S, et al. Experiences of patients with complex needs at municipal emergency outpost satellites. Scand J Prim Health Care. 2025:1–12. doi: 10.1080/02813432.2025.2502095. PMC12632231 40340701

[2] Greenhalgh T, Wherton J, Shaw S, et al. Video consultations for covid-19. BMJ. 2020;368:m998. doi: 10.1136/bmj.m998. 32165352

[3] Bütün A. Emergency department overcrowding in Türkiye. Anatolian J Emerg Med. 2024;7(2):95–96. doi: 10.54996/anatolianjem.1465121.

[4] Scott Kruse C, Karem P, Shifflett K, et al. Evaluating barriers to adopting telemedicine worldwide: a systematic review. J Telemed Telecare. 2018;24(1):4–12. doi: 10.1177/1357633X16674087. 29320966 PMC5768250

[5] Li MM, Rising KL, Goldberg EM. Transitioning to telehealth? A guide to evaluating outcomes. Health Policy Technol. 2022;11(3):100623. doi: 10.1016/j.hlpt.2022.100623. 35369128 PMC8957891

